# Comparison of Corneal Densitometry and Visual Quality after Small Incision Lenticule Extraction (SMILE) and Laser Epithelial Keratomileusis (LASEK): One-Year Comparative Study

**DOI:** 10.1155/2023/3430742

**Published:** 2023-02-02

**Authors:** Wenting Cai, Lin Liu, Min Li, Yuehui Shi, Lina Sun, Xiaoyun Ma, Jun Zou

**Affiliations:** ^1^Department of Ophthalmology, Shanghai Tenth People's Hospital, Tongji University, Shanghai 200072, China; ^2^Department of Ophthalmology, Shanghai University of Medicine & Health Sciences Affiliated Zhoupu Hospital, Shanghai, China

## Abstract

**Purpose:**

To investigate changes in corneal densitometry (CD) and visual quality following small incision lenticule extraction (SMILE) and laser epithelial keratomileusis (LASEK) in patients with mild-to-moderate myopia.

**Methods:**

A retrospective analysis was performed on 24 and 25 patients (46 eyes each) who underwent SMILE and LASEK, respectively, for mild-to-moderate myopia. The visual quality and CD values were recorded. Using the Pentacam Scheimpflug system, CD values were collected in three concentric optical zones at the depths of the anterior, central, and posterior layers. Efficacy, safety, predictability, corneal wavefront aberrations, and QoV scores were measured to evaluate visual quality. A correlation analysis was performed between changes in CD and clinical characteristics.

**Results:**

There were no statistical differences in efficacy and safety indices between the two groups. At 3 months postoperatively, a pronounced reduction in several zones was observed in the LASEK group (*p* < 0.05), whereas no obvious change was observed in the SMILE group. There were obvious changes in the CD values in several zones in the SMILE and LASEK groups (*p* < 0.05) after 1 year. The magnitude of the CD changes in the anterior and central corneal layers was smaller in the SMILE group than in the LASEK group (all *p* < 0.05). Lower HOAs, spherical aberration, and horizontal comas of the anterior and whole corneal surfaces were observed in the SMILE group. QoV scores were similar between the two groups.

**Conclusion:**

CD decreased in the SMILE and LASEK groups after 1 year; there was a smaller reduction in SMILE than in LASEK. SMILE and LASEK did not differ significantly in terms of safety and effectiveness in correcting mild-to-moderate myopia.

## 1. Introduction

Myopia is the most frequent cause of distance impairment and a global public health concern, with an increasing annual prevalence. It is estimated that globally, by 2050, 4758 and 938 million people will have myopia and high myopia, respectively, which would constitute 49.8% and 9.8% of the world population, respectively [1]. Corneal refractive surgery remains the mainstay of ocular refractive correction as the cornea provides three-fourth of ocular refractive power. Corneal refractive surgery has been widely performed to correct myopia and is recognized as an effective, safe, and predictable strategy [2].

Small incision lenticule extraction (SMILE) is an advanced technology of corneal refractive surgery without flap creation, mainly depending on a 3-4 mm side cut for lenticule extraction [3]. Laser epithelial keratomileusis (LASEK) mainly utilizes an excimer laser to correct myopia. Both procedures are safe and effective [4, 5].

Corneal transparency is indispensable for achieving optimal vision. It can be measured via corneal light backscatter and is commonly displayed as corneal densitometry (CD) values [6]. The CD values can be investigated via several medical devices, such as *in vivo* confocal microscopy [7], Oculus Pentacam [8], and optical coherence tomography [9]. The Pentacam Scheimpflug system (Oculus GmbH, Wetzlar, Germany) was used for scanning the cornea and recording CD values. This is a noninvasive and convenient approach for evaluating the backscatter profile of the anterior, central, and posterior layers of the entire cornea. This method has been used to evaluate the progression of various ocular surface diseases, including corneal dystrophies [10] and keratoconjunctivitis [11], and corneal clarity after refractive corneal surgery [12, 13]. Previous studies reported that the CD values increased quickly in the first 24 h after SMILE [14]. Meanwhile, Wei et al. showed that the CD values decreased significantly after SMILE or femtosecond laser-assisted in situ keratomileusis (FS-LASIK) at 5 years, with fewer changes found after SMILE [15]. No studies have compared CD between SMILE and LASEK after a 1-year follow-up. Therefore, we used the Pentacam Scheimpflug system to evaluate CD changes in SMILE and LASEK at baseline, 3 months, and 1 year postoperatively. Our study provided a different evaluating index to compare these two types of refractive surgery and examine the relationship with clinical characteristics.

## 2. Materials and Methods

### 2.1. Demographics

In this retrospective study, we compared 92 eyes of 49 patients who underwent SMILE (23 right and 23 left eyes of 24 patients) or LASEK (22 right and 24 left eyes of 25 patients) at the Department of Ophthalmology, Shanghai Tenth People's Hospital (Shanghai, China), between May 2020 and January 2021. The sample size was calculated using PASS 15.0. All the procedures were performed in accordance with the principles of the Declaration of Helsinki.

### 2.2. Inclusion and Exclusion Criteria

The main inclusion criteria were (1) age ≥ 18 years with stable refraction for at least 2 years, (2) spherical equivalent (SE) < −6.00 D, (3) corrected distance visual acuity of 20/20 or better, and (4) discontinuation for 2 weeks and 6 months of soft contact and orthokeratology lens use, respectively, before examination. The exclusion criteria were a history of ocular disease, surgery, or systemic diseases.

Preoperative examinations included measurement of visual acuity (uncorrected distance visual acuity (UDVA) and corrected distance visual acuity (CDVA)), noncontact intraocular pressure (IOP), refraction (objective, manifest, and cycloplegic), axial length (AL), lenticule thickness (LT), ablation depth (AD), minimum corneal thickness (MCT), corneal curvature, densitometry, corneal wavefront aberrations, and fundoscopy.

### 2.3. Surgical Techniques

All the surgical procedures were performed by a single surgeon (Dr. Zou) with extensive experience. The VisuMax femtosecond laser system (Carl Zeiss Meditec, Jena, Germany) settings were as follows: 500 kHz repetition rate, 150 nJ pulse energy, 120 *μ*m intended cap thickness, and 6.7-6.8 mm optical zone. An incision with a circumferential width of 2-3 mm was made for lenticule dissection and extraction.

LASEK was performed using 20% alcohol solution to create corneal epithelial flaps. The epithelial flaps were lifted using a crescent corneal spatula. The MEL 90 excimer laser platform (Carl Zeiss Meditec AG, Jena, Germany) was used to complete corneal stromal tissue ablation at a repetition rate of 500 Hz.

Postoperatively, topical 0.1% fluorometholone was administered four times daily, tapering off once a week and once a month in the SMILE and LASEK groups, respectively. Topical levofloxacin was administered four times daily for 10 days, and topical sodium hyaluronate was administered four times daily for 3-6 months in both groups.

### 2.4. Corneal Densitometry Analysis

Data on CD values were collected using the Pentacam HR and expressed in grayscale units (GSUs). The values could quantify corneal clarity by monitoring transient haze-like reactions [16]. Four annular zones of the cornea (0-2 mm, 2-6 mm, 6-10 mm, and 10-12 mm) displayed CD values [17]. According to the anatomical corneal layers based on depth, there were four corneal layers, including a superficial anterior layer of 120 *μ*m, a posterior layer of the innermost cornea of 60 *μ*m, central layers of subtraction of the anterior and posterior layer thickness from the total layer thickness, and a total layer [18]. The zone of peripheral 10-12 mm was excluded due to the lowest degree of repeatability and reproducibility of this zone [19].

### 2.5. Corneal Wavefront Aberration Measurement

Corneal wavefront aberrations (anterior, posterior, and whole) were obtained using a Scheimpflug camera system [20]. The analyzed zone was set as a 6 mm diameter around corneal vertex. The root mean square (RMS) values of the total higher-order aberrations (HOAs), spherical aberration (Z4, 0), horizontal coma (Z3, 1), vertical coma (Z3, -1), trefoil 0° (Z3, 3), and trefoil 30° (Z3, -3) were extracted for further analysis.

### 2.6. Quality of Vision (QoV) Questionnaire

The QoV questionnaire was used to measure the subjective visual quality at 1 year postoperatively [21]. There were 10 symptoms scaled by frequency, severity, and bothersome nature, including halos, glare, hazy, starbursts, blurred, double vision, visual fluctuation, distortion, and difficulty in focusing and judging distance or depth perception. The scores for all of the above analyses ranged from 0 to 3 according to the degree. The questionnaires were completed telephonically.

### 2.7. Statistical Analysis

Each eye with mild-to-moderate myopia was selected for the statistical analysis. All data were analyzed using SPSS statistical software (version 26.0, SPSS Inc., Chicago, IL, USA). Continuous variables were presented as mean ± standard deviation (SD), while categorical variables were recorded as counts. The independent *t*-test was used for normally distributed data, and the Mann-Whitney *U* test was used for nonnormally distributed data (except for sex and eye, which were compared using the chi-square test). Analysis of variance was performed in different rounds to evaluate the standardized differences in the preoperative and postoperative visits at different time points. The associations between the changes in CD values and the type of surgery, UDVA, age, SE, endothelial cells, MCT, and LT/AD were assessed using Spearman's correlation analysis. *p* was set at < 0.05.

## 3. Results

### 3.1. Study Population

Among the 49 patients, 46 eyes of 24 patients who underwent SMILE and 46 eyes of 25 patients who underwent LASEK were evaluated at 3 months and 1 year postoperatively. No significant preoperative differences were observed in most clinical characteristics between the two groups (all *p* > 0.05). The MCT and LT/AD were significantly different between the groups before surgery (*p* < 0.001) (Table 1). All surgical procedures were uneventful, and no high intraocular pressure or any other vision-threatening complications were observed after 1 year.

### 3.2. Efficacy and Safety

**Figure 1:**
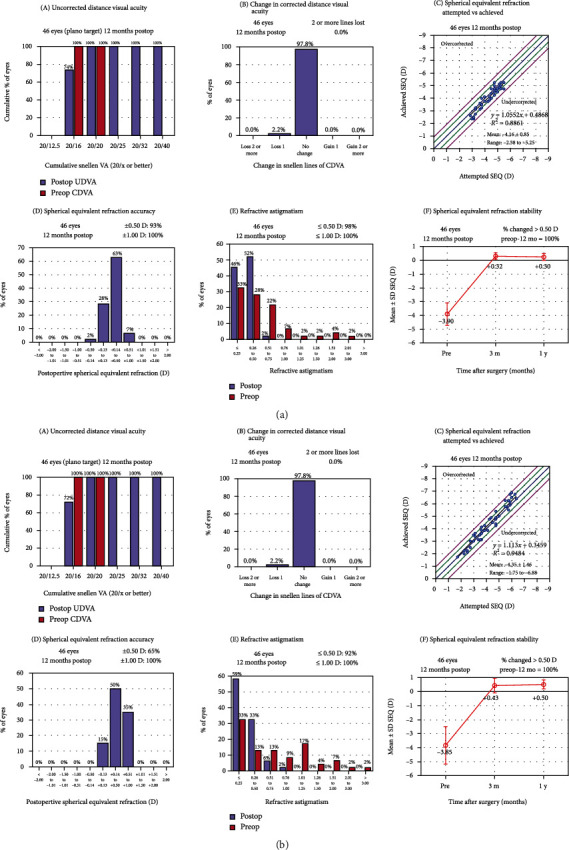
Refractive outcomes at 1 year after the SMILE (a) and LASEK (b) procedures. (A) postoperative UDVA versus preoperative CDVA; (B) change in CDVA; (C) attempted versus achieved SE refraction change in CDVA; (D) SE refraction accuracy; (E) refractive astigmatism accuracy; (F) SE refractive stability.

At the 1-year follow-up, 34 (74%) and 33 (72%) eyes in the SMILE and LASEK groups, respectively, achieved a UDVA of 20/16 (Figures 1(a) A and 1(b) A). Forty-five eyes (97.8%) in both groups had stable CDVA. Only one (2.2%) eye lost one line of CDVA in both groups, whereas no eyes lost two or more lines in both groups (Figures 1(a) B and 1(b) B). The efficacy index was 0.74 ± 0.44 and 0.72 ± 0.46 in the SMILE and LASEK groups, respectively (*p* = 0.82). The safety index was 0.98 ± 0.15 in both groups.

### 3.3. Predictability

After 1 year, the attempted and achieved spherical equivalent refractions were stable in both two groups (Figures 1(a) C and 1(b) C), and 43 (93%) eyes in the SMILE group and 30 (65%) eyes in the LASEK group achieved within ±0.5 D of SE, whereas no eyes failed to achieve ±1.00 D of SE (Figures 1(a) D and 1(b) D). For astigmatism, 45 (98%) eyes in the SMILE group and 42 (92%) eyes in the LASEK group achieved within 0.5 D of astigmatism, whereas all eyes in both groups achieved astigmatism of no more than 1.00 D (Figures 1(a) E and 1(b) E). At 3 months postoperatively, there was no difference in manifest SE between the groups (*p* = 0.19), whereas there was a significant difference at 1 year postoperatively (*p* < 0.001) (Figures 1(a) F and 1(b) F).

### 3.4. Postoperative Corneal Densitometry Analysis

Three months after SMILE, the postoperative CD at each of the three annuli of all layers showed no changes compared to the preoperative values (all *p* > 0.05). At the 1-year follow-up, the CD values at each of the three annuli (0-2 mm and 6-10 mm, *p* < 0.001, and 6-10 mm, *p* = 0.016) of the anterior layer were reduced. The CD values in the 0-2 mm (*p* < 0.001) and 2-6 mm (*p* < 0.001) zones of the central and posterior layers and the total layer were significantly reduced postoperatively (Supplementary Figure S1A-D and S2A-C and Table 2).

At 3 months after LASEK, the CD values at the 0-2 mm and 2-6 mm (both *p* < 0.001) and 6-10 mm zones (*p* = 0.006) of the anterior layer, 0-2 mm zone (*p* = 0.015) of the central layer, and 0-2 mm and 2-6 mm zone (both *p* < 0.001) of the total layer were reduced. There was no difference in the CD values of the three zones of the posterior layer and other zones of the central layer and total layers compared with the baseline (*p* > 0.05). At the 1-year follow-up, the CD values of each of the three annuli of the anterior layer (all *p* < 0.001), central layer (*p* < 0.001 for 0-2 mm and 2-6 mm and *p* = 0.025 for 6–10 mm), and the total layer (*p* < 0.001 for 0-2 mm and 2-6 mm and *p* = 0.001 for 6–10 mm) were reduced. Meanwhile, the CD values at the 0-2 mm and 2-6 mm zones of the posterior layer (both *p* < 0.001) showed an obvious reduction compared to baseline (Supplementary Figure S1E-H and S2D-F and Table 2).

Comparing the changes in CD between the groups, the CD values in the LASEK group showed a more obvious reduction in the three zones of the anterior layer (all *p* < 0.001), the 0-2 mm zone of the central layer (*p* = 0.032), and the total layer (all *p* < 0.001) at the 3-month follow-up. Similarly, a greater reduction in CD was observed in the LASEK group than in the SMILE group in the three zones of the anterior layer (all *p* < 0.001), central layer (*p* = 0.021 for 0-2 mm, *p* < 0.001 for 2-6 mm, and *p* = 0.027 for 6-10 mm), and total layer (*p* < 0.001 for 0-2 mm and 2-6 mm and *p* = 0.002 for 6–10 mm) after 1 year. However, no significant changes in the CD values were observed in the posterior layer between the groups (*p* > 0.05).

### 3.5. Corneal Wavefront Aberrations

Three months postoperatively, HOAs, spherical aberration, and vertical and horizontal coma of the anterior and whole corneal surfaces increased in both groups (all *p* < 0.05). At 1 year postoperatively, there was a significant increase in all values of anterior and whole corneal aberrations, except trefoil 30° in the SMILE group and trefoil 0° in the LASEK group (all *p* < 0.05). Lower HOAs, spherical aberration, and horizontal comas of the anterior and whole corneal surfaces were observed in the SMILE group than in the LASEK group at 3 months and 1 year postoperatively (Table 3, all *p* < 0.05, respectively).

### 3.6. QoV Scores

**Figure 2:**
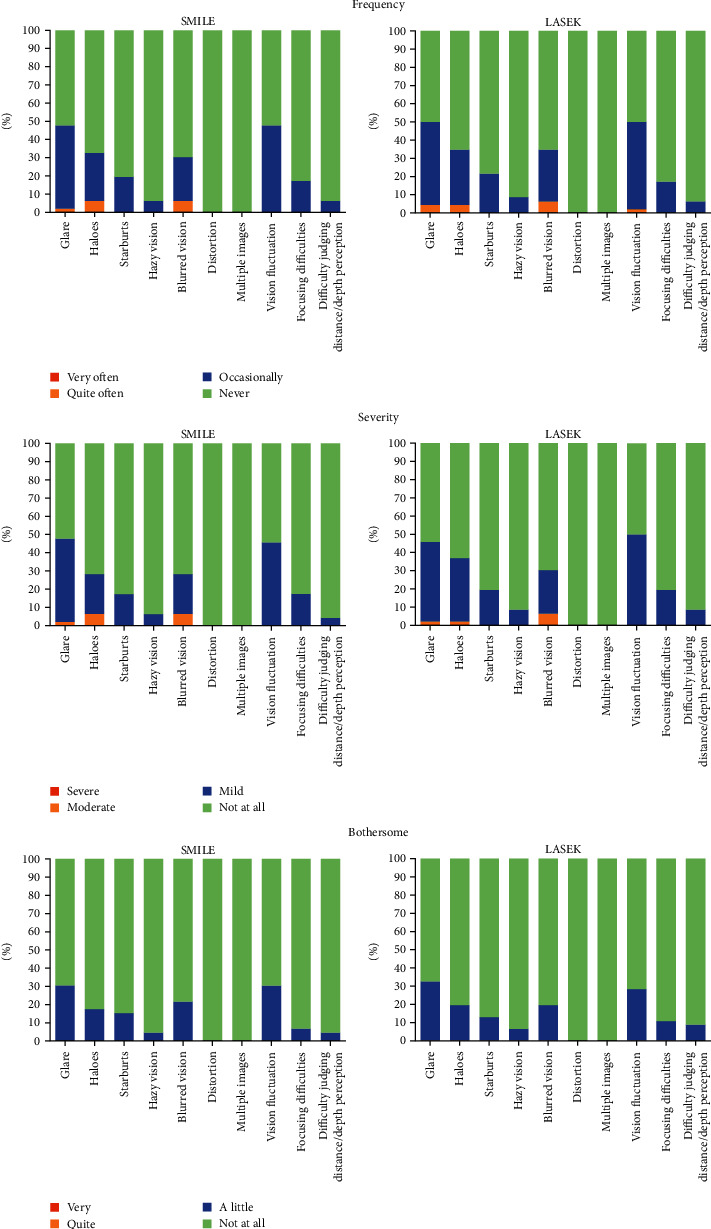
Frequency, severity, and bothersome nature of visual disturbances after SMILE and LASEK surgeries. Bars were ranked in descending order of incidence.

There was no significant difference in the frequency, severity, and bothersome score between the groups at 1 year postoperatively (*p* = 0.72, *p* = 0.56, and *p* = 0.79, respectively) (Figure 2 and Table 4). The three most prevalent reported visual symptoms in the SMILE and LASEK groups were glare (48% versus 50%), vision fluctuation (48% versus 50%), and halos (33% versus 35%). However, most patients considered the severity of their visual symptoms to be mild or nonexistent and responded that it bothered them a little or not at all.

### 3.7. Factors Associated with CD

At 1 year postoperatively, all CD values, except those in the three zones of the posterior corneal layers, were significantly associated with the type of surgery and MCT (*p* < 0.05). Meanwhile, three zones of the anterior layer and one zone of the central and total layers (2-6 mm) were also associated with LT/AD. However, the CD value was not statistically correlated with other characteristics such as age, SE, UCVA, and endothelial cells (Table 5).

## 4. Discussion

In this study, we investigated the changes in CD and visual quality after SMILE and LASEK for mild-to-moderate myopia. A greater reduction in CD was observed in the LASEK group than in the SMILE group in the three zones of all layers, except for the posterior layer, after 1 year. Our findings resonated partly with those of Lazaridis et al. who reported that the CD values recorded by the Scheimpflug showed no difference between baseline and 3 months after SMILE [13]. Rozema et al. indicated that a significant decrease was observed in the anterior zone of corneal backscatter in LASEK surgery at 6 months postoperatively [22]. Furthermore, Litwak et al. also reported good corneal clarity after LASEK [23]. However, Shajari et al. reported that there was no significant difference in CD between LASIK and SMILE at the short- or long-term follow-up [12].

Boote et al. reported that fibril packing over the corneal surface was nonuniform and fibril matrix appeared more compact in the prepupillary cornea [24]. Ni et al. reported that the corneal anterior layer showed higher densitometry than the central and posterior layers [19]. Hence, we hypothesized that the decrease in CD was possibly due to the ablation of the anterior layer. Our results were consistent with those of a previous study performed using confocal microscopy, which suggested that the increased light scatter was correlated with activated keratocytes and mainly occurred in the anterior one-third of the corneal stroma [25]. At the early stage after surgery, corneal transparency increased in response to inflammation, inducing a longer time for the recovery of visual outcomes. Topical steroid eye drops are commonly used to alleviate the inflammatory reactions. Thus, owing to the control of inflammation and ablation of stromal collagen fibrils, a decrease in CD was observed after both SMILE and LASEK 1 year postoperatively. The femtosecond laser in SMILE procedure was applied to create lenticule, accompanied with plasma, shockwave, and cavitation bubble, while the excimer laser in LASEK producer was applied to ablation, followed with the damage of the organic molecular bonds of cornea [26, 27]. Considering this situation, investigating the significance of regional CD in these two surgeries was meaningful.

There was no significant difference in the efficacy or safety index between SMILE and LASEK at 1 year postoperatively, suggesting that LASEK was as safe and effective as SMILE for the treatment of mild-to-moderate myopia. Our findings partly confirmed those of Yu et al. who reported no difference in visual outcomes after SMILE and LASEK for low-to-moderate myopia at 3 months postoperatively [28]. Our previously published studies also proved the safety and stability of LASEK for the correction of mild, moderate, and high myopia [29, 30]. However, SMILE became a superior choice over LASEK in terms of lesser intensity of postoperative pain. Exposure to corneal nerve endings and the release of inflammatory mediators lead to intense postoperative pain after LASEK. The pain was relieved by complete corneal reepithelialization [31]. Moreover, considering patients with thinner corneal thickness who required refractive surgery, LASEK had the advantage of less ablation depth than the lenticule thickness of SMILE for correcting the same SE. Hence, the two surgical procedures have their respective advantages and disadvantages for myopic correction, with almost consistent long-term visual outcomes.

With the development of refractive surgery, increasing attention has been paid to the postoperative visual quality. HOAs induced by corneal refractive surgeries can cause symptoms such as glare, halos, and starbursts [32]. Sekundo et al. found that 54 eyes after SMILE showed a slight increase in HOAs, coma, and spherical aberrations from 3 to 12 months [33]. Previous studies reported that the changes in corneal HOAs and spherical aberration were significantly higher after LASEK than after SMILE from 3 months to 3 years [34]. Our results were also consistent with those observed at 3 months and 1 year in previous studies [5, 28]. QoV is an important indicator for assessing postoperative visual quality and patient satisfaction. Yu et al. indicated that the scores of glare and halos in the QoV questionnaire were lower in the SMILE group than in the LASEK group at the 3-month follow-up [28]. In the present study, there was a slightly lower induction rate of HOAs, spherical aberrations, and horizontal coma in the SMILE group than in the LASEK group after 1 year. Additionally, there was no significant difference in the subjective visual symptoms between the groups postoperatively. The reduction of CD values might influence the actual visual function. A previous study reported that there was no relationship between the changes of CD values with the visual function such as corneal wavefront aberrations [15]. And in our study, we found that the efficacy index, safety index, and QoV scores had no significant difference between SMILE and LASEK group while the CD values (anterior, central, and total layers) were significantly different. Our findings were somewhat similar to those of Lazaridis et al., indicating that CD was associated with LT/AD [13]. In our study, all CD values, except those at the posterior corneal layers, were significantly associated with the type of surgery, MCT, and LT/AD. Since the lenticule thickness in SMILE procedure and the ablation depth in LASEK procedure were applied at the anterior elastic layer and stroma of cornea, the correlation of CD and LT/AD was mainly at the region of anterior layer. This might be associated with the volume of extracted or ablated stromal corneal tissues, inducing the decrease of keratocyte density, so that influenced the backscattered light from residual corneal tissue.

Our study has some limitations. First, the sample size of 46 eyes in each group was relatively small, which increased the risk of a type 2 statistical error (false-negative results because of the underpowered study arms). Therefore, our results were therefore conservative in terms of the differences between the SMILE and LASEK procedures. Second, we mainly analyzed the data at 3-month and 1-year follow-up. Therefore, our study was not equipped to make inferences on longer-term changes in the CD values and visual outcomes between the SMILE and LASEK procedures. Third, the Scheimpflug device showed greater reflection at the interfaces between layers with different refractive indices owing to noncontact examination.

In conclusion, CD decreased in both the SMILE and LASEK groups at the 1-year follow-up; however, the reduction in CD values in the SMILE group was smaller than that in the LASEK group. Nevertheless, SMILE and LASEK are equally safe, effective, and predictable refractive surgeries for correcting mild-to-moderate myopia. Long-term observations and mechanisms of CD changes after SMILE and LASEK should be employed in further research.

## Figures and Tables

**Table 1:** Characteristics of the study groups.

Characteristics	SMILE group (*n* = 46 eyes)		LASEK group (*n* = 46 eyes)		*p* value
	Mean ± SD	Range	Mean ± SD	Range	
Age (mean ± SD, year)	28.21 ± 6.10	19 to 40	30.20 ± 7.66	19 to 45	0.320
Sex (male/female)	8/16	—	10/15	—	0.630
Eye (R/L)	23/23	—	22/24	—	0.835
Preoperative spherical error (D)	−3.61 ± 0.84	-5.00 to -1.75	−3.45 ± 1.43	-5.75 to -1.25	0.506
Preoperative cylinder (D)	−0.57 ± 0.58	-2.50 to 0.00	−0.79 ± 0.78	-3.75 to 0.00	0.133
Preoperative SE (D)	−3.90 ± 0.81	-5.00 to -2.13	−3.85 ± 1.34	-5.88 to -1.25	0.815
UDVA (logMAR)	1.30 ± 0.24	0.70 to 1.60	1.21 ± 0.32	0.54 to 1.60	0.107
CDVA (logMAR)	−0.10 ± 0.00	-0.10 to -0.10	−0.10 ± 0.00	-0.10 to -0.10	1.000
IOP (mmHg)	15.25 ± 2.43	10.00 to 20.00	14.57 ± 2.28	9.60 to 19.40	0.173
Axial length (mm)	25.39 ± 0.81	23.61 to 26.67	25.31 ± 1.09	22.72 to 27.61	0.685
K_1_f (D)	42.78 ± 1.53	40.10 to 45.50	42.41 ± 1.54	40.00 to 46.10	0.254
K_2_f (D)	43.83 ± 1.67	41.30 to 47.90	43.68 ± 1.91	40.70 to 49.30	0.676
K_1_b (D)	−6.15 ± 0.25	-5.70 to -6.60	−6.07 ± 0.25	-5.70 to -6.70	0.155
K_2_b (D)	−6.45 ± 0.29	-6.00 to -7.20	−6.39 ± 0.30	-6.00 to -7.30	0.348
Preoperative MCT (*μ*m)	534.91 ± 20.16	496 to 578	512.39 ± 31.18	472 to 599	<0.001^∗^
Endothelial cell count (/mm^2^)	2857.67 ± 302.85	2121.3 to 3690.1	2828.42 ± 285.86	2273.8 to 3656.8	0.635
LT/AD (*μ*m)	96.48 ± 13.57	71 to 122	72.43 ± 20.26	22 to 102	<0.001^∗^

SMILE: small incision lenticule extraction; LASEK: laser epithelial keratomileusis; SD: standard deviation; D: diopters; SE: spherical equivalent; UDVA: uncorrected distance visual acuity; CDVA: corrected distance visual acuity; IOP: intraocular pressure; MCT: minimum corneal thickness; LT: lenticule thickness for SMILE patients; AD: ablation depth for LASEK patients; ^∗^*p* < 0.05, significant difference between the SMILE and LASEK groups.

**Table 2:** Comparison of corneal densitometry (CD) between the SMILE and LASEK groups.

CD (GSU)		Anterior layer			Central layer			Posterior layer			Total thickness		
		0-2 mm	2-6 mm	6-10 mm	0-2 mm	2-6 mm	6-10 mm	0-2 mm	2-6 mm	6-10 mm	0-2 mm	2-6 mm	6-10 mm
Preoperative	SMILE	22.95 ± 1.44	20.61 ± 1.34	20.03 ± 3.43	13.7 ± 0.85	12.25 ± 0.64	12.73 ± 1.88	10.83 ± 0.84	9.94 ± 0.57	11.03 ± 1.39	15.89 ± 0.99	14.26 ± 0.74	14.6 ± 2.17
	LASEK	23.48 ± 1.15	20.92 ± 0.98	20.61 ± 3.84	13.89 ± 0.58	12.42 ± 0.49	12.86 ± 2	10.78 ± 0.87	9.89 ± 0.71	11.13 ± 1.58	16.05 ± 0.7	14.4 ± 0.57	14.86 ± 2.37
	*P-value*	0.053	0.201	0.451	0.209	0.157	0.764	0.807	0.687	0.743	0.375	0.316	0.583

Postoperative month 3	SMILE	22.51 ± 1.33	20.6 ± 1.1	19.77 ± 3.43	13.73 ± 0.77	12.5 ± 0.63	13 ± 2.02	10.5 ± 0.78	10.07 ± 0.65	11.51 ± 1.58	15.57 ± 0.82	14.39 ± 0.67	14.76 ± 2.26
	Change	-0.44 ± 1.32	0 ± 1.23	-0.26 ± 1.75	0.04 ± 0.88	0.25 ± 0.71	0.26 ± 0.9	-0.33 ± 0.99	0.13 ± 0.83	0.48 ± 0.81	-0.32 ± 0.98	0.13 ± 0.8	0.16 ± 1.1
	LASEK	20.13 ± 1.28	18.22 ± 1.25	18.4 ± 3.69	14.28 ± 0.9	12.64 ± 0.62	12.83 ± 1.88	10.74 ± 0.71	10.05 ± 0.57	11.4 ± 1.47	15.05 ± 0.81	13.63 ± 0.66	14.21 ± 2.27
	Change	-3.36 ± 1.27^#^	-2.7 ± 1.16^#^	-2.2 ± 1.36^#^	0.39 ± 0.66^#^	0.22 ± 0.45	-0.02 ± 0.7	-0.05 ± 1.05	0.16 ± 0.83	0.27 ± 0.73	-1.01 ± 0.78^#^	-0.77 ± 0.63^#^	-0.65 ± 0.82
	*Δ*change	-2.92 ± 1.79^†^	-2.7 ± 1.72^†^	-1.94 ± 2.37^†^	0.35 ± 1.03^†^	-0.03 ± 0.77	-0.28 ± 1.08	0.28 ± 1.34	0.03 ± 1.17	-0.21 ± 1.04	-0.68 ± 1.21^†^	-0.9 ± 0.97^†^	-0.81 ± 1.35^†^
	95% CI	(-3.45, -2.38)	(-3.19, -2.20)	(-2.59, -1.29)	(0.03, 0.67)	(-0.27, 0.22)	(-0.62, 0.05)	(-0.14, 0.71)	(-0.32, 0.37)	(-0.53, 0.11)	(-1.05, -0.32)	(-1.19, -0.60)	(-1.21, -0.41)

Postoperative year 1	SMILE	20.01 ± 1.3	18.64 ± 1.07	18.38 ± 3.05	12.47 ± 0.68	11.65 ± 0.53	12.29 ± 1.76	9.55 ± 0.74	9.39 ± 0.59	10.85 ± 1.39	14 ± 0.81	13.23 ± 0.64	13.84 ± 1.99
	Change	-2.94 ± 1.19^∗^	-1.97 ± 0.99^∗^	-1.66 ± 2.61^∗^	-1.23 ± 0.62^∗^	-0.6 ± 0.52^∗^	-0.44 ± 1.21	-1.28 ± 0.99^∗^	-0.55 ± 0.82^∗^	-0.18 ± 0.91	-1.89 ± 0.8^∗^	-1.04 ± 0.67^∗^	-0.76 ± 1.5
	LASEK	17.87 ± 0.98	16.51 ± 0.92	17.13 ± 3.82	12.35 ± 0.48	11.37 ± 0.36	11.91 ± 1.98	9.45 ± 0.63	9.1 ± 0.5	10.6 ± 1.53	13.23 ± 0.57	12.32 ± 0.44	13.22 ± 2.36
	Change	-5.61 ± 1.31^#^	-4.41 ± 1.04^#^	-3.48 ± 1.71^#^	-1.54 ± 0.65^#^	-1.05 ± 0.47^#^	-0.95 ± 0.9^#^	-1.34 ± 1.02^#^	-0.79 ± 0.79^#^	-0.53 ± 0.81	-2.82 ± 0.83^#^	-2.08 ± 0.61^#^	-1.64 ± 1.06^#^
	*Δ*change	-2.67 ± 1.87^†^	-2.45 ± 1.3^†^	-1.82 ± 3.07^†^	-0.31 ± 0.79^†^	-0.45 ± 0.59^†^	-0.5 ± 1.51^†^	-0.06 ± 1.41	-0.24 ± 1.14	-0.35 ± 1.21	-0.93 ± 1.09^†^	-1.04 ± 0.77^†^	-0.88 ± 1.8^†^
	95% CI	(-3.19, -2.15)	(-2.87, -2.03)	(-2.74, -0.91)	(-0.57, -0.05)	(-0.65, -0.24)	(-0.95, -0.06)	(-0.48, 0.36)	(-0.57, 0.10)	(-0.71, 0.01)	(-1.27, -0.59)	(-1.31, -0.78)	(-1.42, -0.34)

^||^ *p* < 0.05, significant difference in preoperative values between the SMILE and LASEK groups. ^∗^*p* < 0.05, significantly different from the preoperative values in the SMILE group. ^#^*p* < 0.05, significantly different from the preoperative values in the LASEK group. ^†^*p* < 0.05, greater change in the LASEK group than in the SMILE group. *Δ*Change = difference in the changes between the two groups; CI = confidence interval; GSU = grayscale units.

**Table 3:** Corneal wavefront aberrations at the front, back, and total layers in the SMILE and LASEK groups preoperatively and at 3 months and 1 year postoperatively.

Wavefront aberrations	Preoperative			Postoperative month 3			Postoperative year 1		
	SMILE	LASEK	*p*	SMILE	LASEK	*p*	SMILE	LASEK	*p*
*Front cornea*									
HOAs	0.37 ± 0.06	0.37 ± 0.08	0.96	0.61 ± 0.15^∗^	0.70 ± 0.25^#^	0.02^†^	0.63 ± 0.14^∗^	0.72 ± 0.21^#^	0.02^†^
Spherical aberration	0.24 ± 0.07	0.21 ± 0.07	0.06	0.33 ± 0.1^∗^	0.48 ± 0.20^#^	<0.001^†^	0.35 ± 0.10^∗^	0.47 ± 0.16^#^	<0.001^†^
Z (3, -1)	0.11 ± 0.07	0.14 ± 0.10	0.07	0.30 ± 0.19^∗^	0.22 ± 0.16^#^	0.03^†^	0.31 ± 0.22^∗^	0.22 ± 0.16^#^	0.03^†^
Z (3, 1)	0.11 ± 0.08	0.13 ± 0.08	0.32	0.22 ± 0.15^∗^	0.27 ± 0.25^#^	0.19	0.22 ± 0.15^∗^	0.31 ± 0.25^#^	0.048^†^
Z (3, -3)	0.10 ± 0.07	0.07 ± 0.06	0.06	0.12 ± 0.11	0.09 ± 0.08	0.18	0.10 ± 0.10	0.12 ± 0.08^#^	0.27
Z (3, 3)	0.06 ± 0.04	0.07 ± 0.05	0.38	0.07 ± 0.06	0.09 ± 0.08	0.13	0.08 ± 0.06^∗^	0.08 ± 0.06	0.90
*Back cornea*									
HOAs	0.19 ± 0.03	0.18 ± 0.03	0.51	0.19 ± 0.03	0.19 ± 0.04	0.57	0.19 ± 0.03	0.19 ± 0.03	0.65
Spherical aberration	0.16 ± 0.02	0.15 ± 0.02	0.28	0.16 ± 0.02	0.15 ± 0.02	0.14	0.16 ± 0.03	0.16 ± 0.02	0.28
Z (3, -1)	0.02 ± 0.02	0.03 ± 0.02	0.10	0.03 ± 0.02	0.03 ± 0.02	0.91	0.03 ± 0.02	0.03 ± 0.02	0.53
Z (3, 1)	0.02 ± 0.01	0.02 ± 0.01	0.85	0.02 ± 0.02	0.02 ± 0.02	0.91	0.02 ± 0.02	0.02 ± 0.02	0.81
Z (3, -3)	0.03 ± 0.02	0.03 ± 0.02	0.08	0.04 ± 0.03	0.03 ± 0.03	0.35	0.04 ± 0.03	0.04 ± 0.03	0.69
Z (3, 3)	0.03 ± 0.03	0.04 ± 0.03	0.73	0.04 ± 0.03	0.04 ± 0.04	0.73	0.03 ± 0.03	0.03 ± 0.02	0.63
*Total cornea*									
HOAs	0.35 ± 0.07	0.36 ± 0.09	0.62	0.63 ± 0.16^∗^	0.73 ± 0.26^#^	0.03^†^	0.65 ± 0.15^∗^	0.74 ± 0.23^#^	0.02^†^
Spherical aberration	0.18 ± 0.07	0.16 ± 0.07	0.06	0.29 ± 0.11^∗^	0.45 ± 0.22^#^	<0.001^†^	0.31 ± 0.11^∗^	0.44 ± 0.17^#^	<0.001^†^
Z (3, -1)	0.11 ± 0.07	0.14 ± 0.09	0.06	0.32 ± 0.20^∗^	0.23 ± 0.17^#^	0.02^†^	0.34 ± 0.23^∗^	0.23 ± 0.19^#^	0.01^†^
Z (3, 1)	0.11 ± 0.08	0.13 ± 0.09	0.25	0.23 ± 0.16^∗^	0.29 ± 0.26^#^	0.17	0.23 ± 0.16^∗^	0.33 ± 0.26^#^	0.04^†^
Z (3, -3)	0.10 ± 0.07	0.08 ± 0.07	0.26	0.13 ± 0.13	0.11 ± 0.10	0.48	0.12 ± 0.10	0.15 ± 0.1^#^	0.23
Z (3, 3)	0.06 ± 0.05	0.07 ± 0.05	0.28	0.07 ± 0.06	0.09 ± 0.09	0.21	0.09 ± 0.07^∗^	0.09 ± 0.07	0.99

^∗^ *p* < 0.05, significantly different from the preoperative values in the SMILE group. ^#^*p* < 0.05, significantly different from the preoperative values in the LASEK group. ^†^*p* < 0.05, significant difference between the SMILE and LASEK groups.

**Table 4:** Postoperative QoV scores after SMILE and LASEK at the 1-year follow-up.

QoV score	SMILE	LASEK	*p* value
*Frequency*			0.72
Mean ± SD	2.24 ± 2.04	2.39 ± 2.03	
Median	2	2	
Range	0 to 7	0 to 7	
IQR	0 to 6	0 to 6	
*Severity*			0.56
Mean ± SD	2.11 ± 1.96	2.35 ± 2	
Median	2	2	
Range	0 to 7	0 to 7	
IQR	0 to 6	0 to 6	
*Bothersome*			0.79
Mean ± SD	1.3 ± 1.68	1.39 ± 1.39	
Median	1	1	
Range	0 to 6	0 to 6	
IQR	0 to 6	0 to 6	

SMILE: small incision lenticule extraction; LASEK: laser epithelial keratomileusis; SD: standard deviation; IQR: interquartile range.

**Table 5:** Univariate analysis between the change of corneal densitometry after refractive surgery and type of operation, age, SE, UCVA, endothelial cell, MCT, and LT/AD.

Variable	Postoperative year 1													
	Group		Age		SE		UCVA		Endothelial cell		MCT		LT/AD	
	*r*	*p*	*r*	*p*	*r*	*p*	*r*	*p*	*r*	*p*	*r*	*p*	*r*	*p*
Anterior layer														
0-2 mm	-0.767	<0.001^∗^	-0.089	0.399	0.125	0.233	-0.057	0.592	-0.049	0.643	0.434	<0.001^∗^	0.316	0.002^∗^
2-6 mm	-0.812	<0.001^∗^	-0.065	0.539	0.054	0.606	-0.088	0.404	-0.001	0.995	0.537	<0.001^∗^	0.407	<0.001^∗^
6-10 mm	-0.448	<0.001^∗^	-0.020	0.849	0.018	0.864	0.010	0.927	-0.108	0.307	0.247	0.018^∗^	0.244	0.019^∗^
Central layer														
0-2 mm	-0.243	0.019^∗^	0.102	0.331	0.168	0.109	0.052	0.623	0.006	0.958	0.408	<0.001^∗^	0.022	0.837
2-6 mm	-0.423	<0.001^∗^	0.045	0.669	-0.005	0.961	-0.020	0.849	-0.068	0.520	0.454	<0.001^∗^	0.224	0.032^∗^
6-10 mm	-0.213	0.041^∗^	-0.016	0.877	-0.041	0.701	0.074	0.484	-0.128	0.225	0.222	0.033^∗^	0.160	0.128
Posterior layer														
0-2 mm	-0.060	0.571	0.203	0.052	0.038	0.721	0.156	0.138	0.117	0.265	0.130	0.217	0.030	0.777
2-6 mm	-0.147	0.163	0.188	0.072	-0.054	0.612	0.065	0.537	0.137	0.194	0.173	0.099	0.133	0.206
6-10 mm	-0.182	0.082	0.118	0.262	-0.013	0.902	0.106	0.313	-0.021	0.842	0.132	0.211	0.145	0.169
Total layer														
0-2 mm	-0.510	<0.001^∗^	0.088	0.405	0.135	0.201	0.031	0.770	0.033	0.754	0.407	<0.001^∗^	0.201	0.055
2-6 mm	-0.668	<0.001^∗^	0.049	0.641	0.031	0.766	-0.056	0.596	0.040	0.702	0.510	<0.001^∗^	0.350	0.001^∗^
6-10 mm	-0.347	0.001^∗^	0.035	0.741	-0.006	0.951	0.047	0.658	-0.097	0.357	0.250	0.016^∗^	0.205	0.050

SE: spherical equivalent; LT/AD: lenticule thickness/ablation depth; MCT: minimum corneal thickness; UCVA: uncorrected distance visual acuity.

## Data Availability

The data used to support the findings of this study are included within the article.

## Supplementary Materials

Supplementary Materials Supplementary Figure S1: time course of corneal densitometry in the SMILE and LASEK groups. (A) SMILE and (B) LASEK groups. ^∗^*p* < 0.05, significantly different from the preoperative values in the SMILE and LASEK groups. Supplementary Figure S2: diagram of changes of CD values of three layers after SMILE and LASEK. (A–C) Diagram of changes in CD relative to baseline (red represents a statistically significant reduction) in the SMILE group. (D, E) Diagram of the changes in CD relative to baseline (purple represents a statistically significant reduction) in the LASEK group. Left semicircle: postoperative month 3; right semicircle: postoperative year 1.
