# Supplementary material for: Comparison of Corneal Densitometry and Visual Quality after Small Incision Lenticule Extraction (SMILE) and Laser Epithelial Keratomileusis (LASEK): One-Year Comparative Study
Source: Biomed Res Int. 2023 Feb 2;2023:3430742. doi: 10.1155/2023/3430742 (PMC9911227; doi:10.1155/2023/3430742)
Supplement: Supplementary Materials — Supplementary Figure S1: time course of corneal densitometry in the SMILE and LASEK groups. (A) SMILE and (B) LASEK groups. ∗p < 0.05, significantly different from the preoperative values in the SMILE and LASEK groups. Supplementary Figure S2: diagram of changes of CD values of three layers after SMILE and LASEK. (A–C) Diagram of changes in CD relative to baseline (red represents a statistically significant reduction) in the SMILE group. (D, E) Diagram of the changes in CD relative to baseline (purple represents a statistically significant reduction) in the LASEK group. Left semicircle: postoperative month 3; right semicircle: postoperative year 1. [file 3430742.f1.pdf]

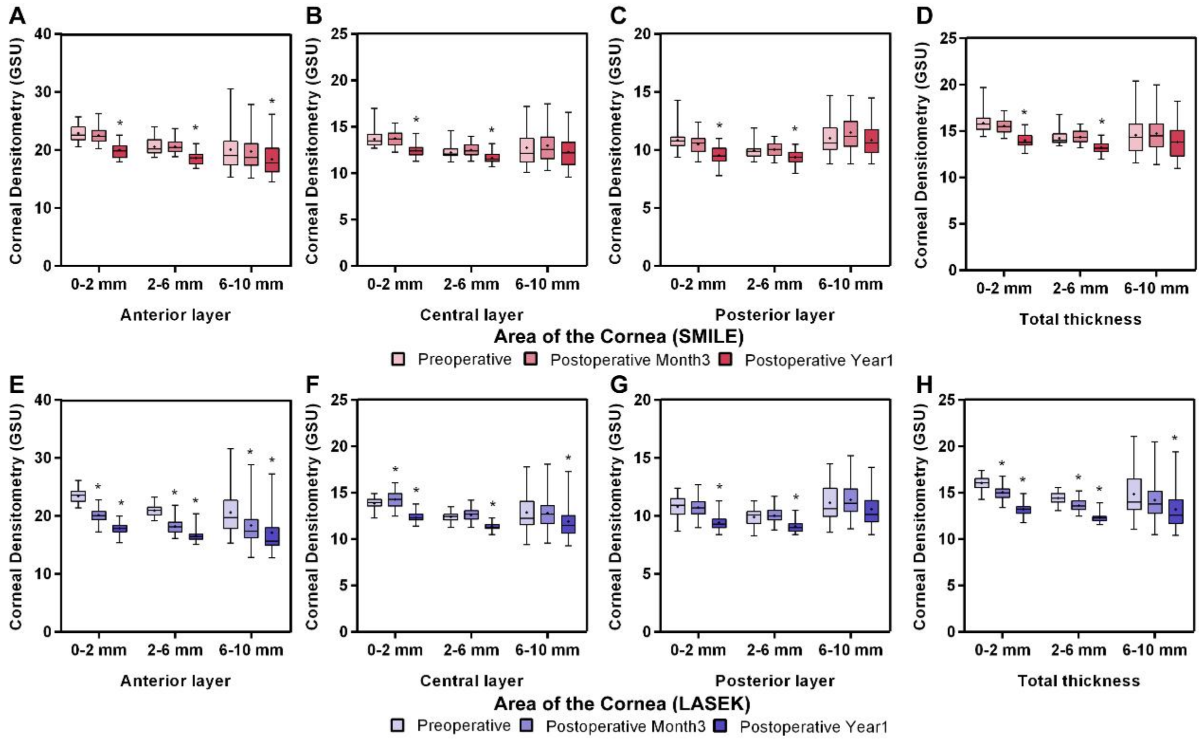

Supplementary Figure S1: Time course of corneal densitometry in the SMILE and LASEK groups. (A) SMILE and (B) LASEK groups. *$p < 0.05$, significantly different from the preoperative values in the SMILE and LASEK groups.

SMILE: small incision lenticule extraction, LASEK: laser epithelial keratomileusis

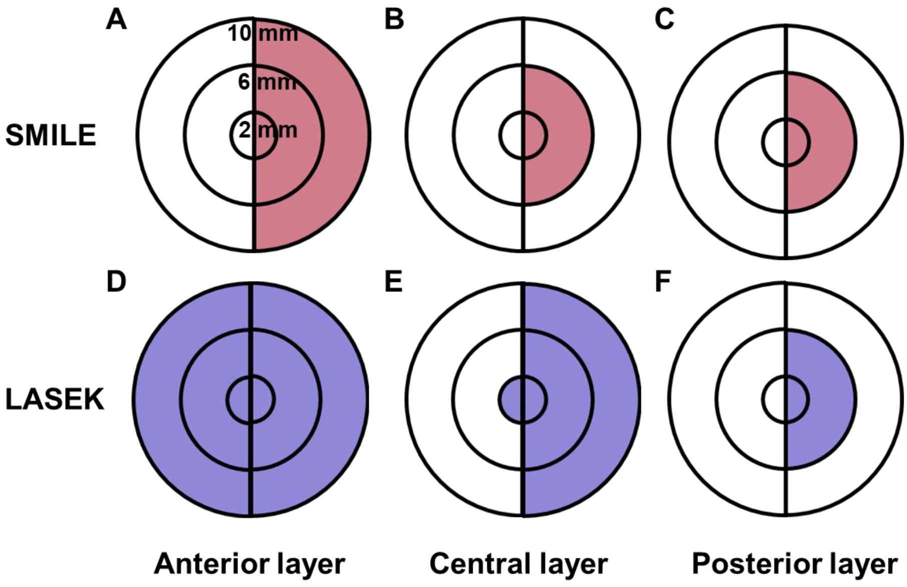

Supplementary Figure S2: Diagram of changes of CD values of three layers after SMILE and LASEK. (A-C) Diagram of changes in CD relative to baseline (red represents a statistically significant reduction) in the SMILE group. (D-E) Diagram of the changes in CD relative to baseline (purple represents a statistically significant reduction) in the LASEK group. Left semicircle: postoperative month 3; Right semicircle: postoperative year 1.

SMILE: small incision lenticule extraction, LASEK: laser epithelial keratomileusis, CD: corneal densitometry
